# A genome-wide survey of copy number variations reveals an asymmetric evolution of duplicated genes in rice

**DOI:** 10.1186/s12915-020-00798-0

**Published:** 2020-06-26

**Authors:** Fengli Zhao, Yuexing Wang, Jianshu Zheng, Yanling Wen, Minghao Qu, Shujing Kang, Shigang Wu, Xiaojuan Deng, Kai Hong, Sanfeng Li, Xing Qin, Zhichao Wu, Xiaobo Wang, Cheng Ai, Alun Li, Longjun Zeng, Jiang Hu, Dali Zeng, Lianguang Shang, Quan Wang, Qian Qian, Jue Ruan, Guosheng Xiong

**Affiliations:** 1 Shenzhen Branch, Guangdong Laboratory for Lingnan Modern Agriculture; Genome Analysis Laboratory of the Ministry of Agriculture, Agricultural Genomics Institute at Shenzhen, Chinese Academy of Agricultural Sciences, Shenzhen, 518120 China; 2 State Key Laboratory of Rice Biology, China National Rice Research Institute, Hangzhou, 310006 China; 3 Key Laboratory of Genomic and Precision Medicine, Beijing Institute of Genomics, Chinese Academy of Sciences, Beijing, 100101 China; 4 Plant Phenomics Research Center, Nanjing Agricultural University, Nanjing, 210095 China

**Keywords:** Copy number variation, Gene expression, Duplicated gene, Evolutionary fate, Asymmetric evolution

## Abstract

**Background:**

Copy number variations (CNVs) are an important type of structural variations in the genome that usually affect gene expression levels by gene dosage effect. Understanding CNVs as part of genome evolution may provide insights into the genetic basis of important agricultural traits and contribute to the crop breeding in the future. While available methods to detect CNVs utilizing next-generation sequencing technology have helped shed light on prevalence and effects of CNVs, the complexity of crop genomes poses a major challenge and requires development of additional tools.

**Results:**

Here, we generated genomic and transcriptomic data of 93 rice (*Oryza sativa* L.) accessions and developed a comprehensive pipeline to call CNVs in this large-scale dataset. We analyzed the correlation between CNVs and gene expression levels and found that approximately 13% of the identified genes showed a significant correlation between their expression levels and copy numbers. Further analysis showed that about 36% of duplicate pairs were involved in pseudogenetic events while only 5% of them showed functional differentiation. Moreover, the offspring copy mainly contributed to the expression levels and seemed more likely to become a pseudogene, whereas the parent copy tended to maintain the function of ancestral gene.

**Conclusion:**

We provide a high-accuracy CNV dataset that will contribute to functional genomics studies and molecular breeding in rice. We also showed that gene dosage effect of CNVs in rice is not exponential or linear. Our work demonstrates that the evolution of duplicated genes is asymmetric in both expression levels and gene fates, shedding a new insight into the evolution of duplicated genes.

## Background

Natural variations are the basis of genetic diversity and genome evolution. The detection of natural variations and evaluation of their genetic effects are the keys to understand and interpret the formation of biological phenotypes. Natural variations generally include single nucleotide polymorphisms (SNPs), small InDels (no more than 50 bp), and structural variations (SVs). Copy number variations (CNVs), including deletion and duplication, typically ranged from 1 kb to several Mb [1], are important source of structural variations [2–4]. Many methods have been developed to detect CNV, such as fluorescence in situ hybridization (FISH), quantitative polymerase chain reaction (qPCR), and microarray. However, these methods are not suitable to detect CNVs in natural population, due to the low throughput or the low resolution and sensitivity. With the advantages of next-generation sequencing (NGS) technologies, new approaches and algorithms have been developed to detect novel CNVs in recent years [5, 6]. These methods are mainly based on the individual or combination of the following strategies: read-pair (RP), split read (SR), read depth (RD), de novo assembly (AS) [7–9]. The complexity of crop genomes and the structure and distribution of CNVs, make it a challenge to comprehensively and accurately detect CNVs among different germplasms of crop.

The CNVs occurred in the regulatory sequence region will change the gene expression of their flanking regions; nevertheless, the CNVs occurred in the gene region usually show the dosage effect on gene expression, thus affecting the biological phenotype. The dosage effect of CNVs was more obviously observed in human [10–13], and mice [14], as genome-wide analysis suggested that 85–95% of detected CNVs were associated with changes in gene expression [10, 14]. However, very few genome-wide analyses of CNVs [15–20] and only a few examples of CNVs contributing to phenotypic variation [21–27] have been reported in crops, but these works were mainly focused on the biological function of a single CNV. Therefore, a large-scale CNV data set with high accuracy will be beneficial to understand the dynamic of genome evolution, provide an insight into the genetic basis of important agricultural traits, and contribute to the crop breeding in the future.

Here, we reported a large-scale analysis of the correlation between CNVs and gene expression levels and revealed CNV’s contribution to genetic diversity of germplasms in rice. We generated genomic and transcriptomic data of 93 accessions of rice and developed a new pipeline, which could comprehensively detect genome-wide CNVs with high accuracy. The correlation analysis between gene copy number and expression level found that approximately 13.1% of genes showed significant correlations. Moreover, the analysis of the expression levels and evolutionary fates of different copies revealed an asymmetric evolution of duplicated genes.

## Results

### Detection of copy number variations in 93 rice accessions

**Fig. 1.**
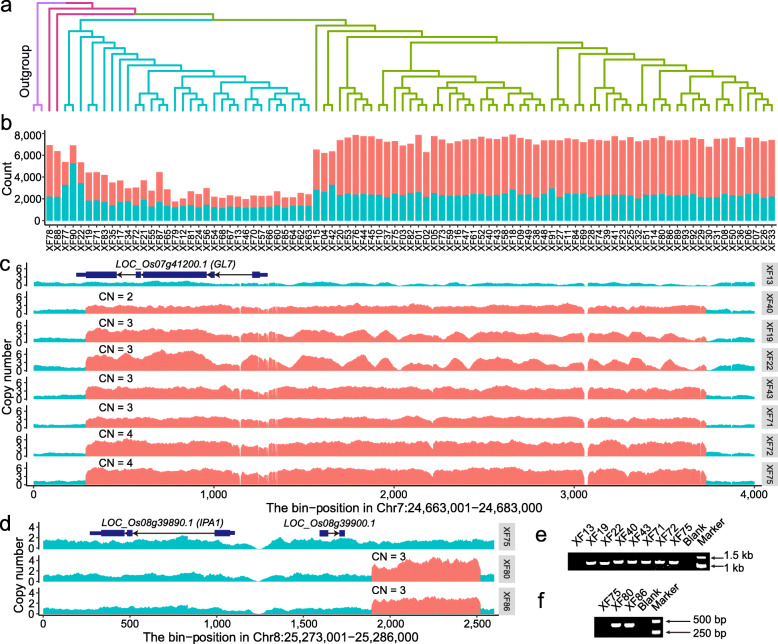
The result and verification of CNV calling for the 93 rice accessions. **a** The phylogenetic tree of the 93 *O. sativa* accessions based on SNP markers, with two *O. glumaepatula* accessions (W1183 and W1187, purple branches) used as outgroup. And *O. sativa* Xian group and Geng group were marked yellowgreen and blue, respectively. The red branches represent two tropical *O. sativa* accessions from Southeast Asia. **b** Number of deletions (red) and duplications (blue) of each accession compared with the Nipponbare RefSeq. **c**, **d** The depth distribution around *GL7* (**c**) and the promotor of *IPA1* (**d**). The red and blue bars showed the duplicated and normal regions, respectively. Each bin represents a length of 5 bp. And XF13 and XF75 were selected as negative controls. **e**, **f** The PCR verification of the duplications around *GL7* (**c**) and the promotor of *IPA1* (**d**)

A total of 93 rice accessions including representative landraces and modern cultivars (Additional file 1: Table S1, Fig. 1a, b) were selected for whole-genome resequencing with average depths about 50× and generated a total of 2.06 Tb of raw reads. Using the Nipponbare RefSeq [28] (version 7.0) as reference, the coverage of these accessions’ resequencing data ranged from 82.81% to 96.06%. The rice root samples grown in hydroponic culture for 35 days were collected for RNA-Seq. The data volume of each sample was above 5 Gb (ranged from 5.03 to 9.86 Gb) and 576 Gb raw RNA-seq data were generated from the 93 accessions in total. The rate of uniquely mapped reads ranged from 79.64% to 90.95% (Additional file 1: Table S1).

To call CNVs in genomes, we integrated CNVnator [29] as well as Delly [30] and developed a new algorithm (named as CtgRef-CNV), which combined read depth and de novo assembly methods (Additional file 2: Figure S1). We firstly assembled the genome of each accession by CtgRef-CNV and then mapped the NGS reads from each accession to its own assembled genome to obtain the depth data. Based on these depth data and alignment results (copy number of each alignment block) between the query genome and the reference genome, we calculated the depth of each fragment of the reference genome, which were used to call CNVs. We then determined the boundary of a CNV using a similar strategy of CNVnator [29] (see “Methods”). By mapping reads to its own genome, the CtgRef-CNV reduces the potential mapping bias due to the difference between a query genome and the reference genome. In addition, the CtgRef-CNV uses the transformed depth data to call CNVs, which could avoid the disadvantages of the assembly-based method in detecting multi-copy regions with few sequence differences. Then we filtered the CNVs detected by these three methods with strict standards to obtain final CNV data set of each sample. In our filter standards, the read depth, split reads, and discordant read pairs were taken into account. For duplications, the percentage of high-depth areas should be no less than 50%. For deletions, the coverage should be no more than 50%, and the number of the split reads/discordant read pairs within the upstream and downstream 500 bp of the two breakpoints should be no less than 5 (For more details see “Methods”).

The de novo assembled genome sizes of 93 accessions ranged from 317.1 to 406.8 Mb, with contig N50 sizes of 85 accessions larger than 10 kb. The BUSCO results showed the genome completeness of 89 accessions was higher than 90% (Additional file 1: Table S1). To uncover the CNVs landscape among different rice accessions, we called CNVs from these samples using CNVnator, Delly, and our CtgRef-CNV. Compared with the Nipponbare reference genome, the CNV number in the genome of each accession varied from 2000 to 8000 (Fig. 1b), which showed there were significant genomic differences between the rice subspecies. The number of CNVs in *Oryza sativa indica* (Xian) group [31] was significantly higher than that in *O. sativa japonica* (Geng) group [31] (Fig. 1a, b, and Additional file 2: Figure S3a, *P* value < 2.2 × 10^− 16^), suggesting that the number of variations within subspecies was less than that within subspecies in terms of CNV, which is consistent with the SV results in the Zhang et al. [32] and Zhou et al. [33]. For each 500-kb non-overlap window in the Nipponbare reference genome, more than 7 CNVs on average were detected (Additional file 2: Figure S2), and the distribution of CNVs on either different chromosomes or different chromosome regions was not even (Additional file 2: Figure S2, and Additional file 2: Figure S3c). We detected a total of 32,051 CNVs (Additional file 3: Table S2) from these 93 accessions, of which 120 CNVs were larger than 100 kb. The length showed a typical L-shaped distribution (Additional file 2: Figure S3b).

### The validation of CNVs

Several CNVs had been found to be associated with variations of important agronomic traits in rice. For example, we detected duplications harbored *GL7* (*LOC_Os07g41200*) [23] in seven accessions, and duplication events occurred at the promoter of *IPA1* (*LOC_Os08g39890*) [25] in two accessions, respectively (Additional file 4: Table S3). All these duplication events were confirmed by PCR experiments (Fig. 1c–f). To further evaluate the validity of our CNV results, 10 random CNV loci were verified in 15 accessions by qPCR (see “Methods”). According to the experimental results, the accuracy of our CNVs was nearly 95% (Additional file 2: Figure S4 and Additional file 4: Table S4). These results indicated that our method was of high accuracy. Moreover, in the phylogenetic tree analysis and principal component analysis (PCA) based on CNVs, the two subspecies are both essentially separated, which are in accordance with the phylogenetic result of SNPs (Additional file 2: Figure S5). In addition, we found that the genes with an extreme high copy number (no less than 10) in more than 5 accessions were common multi-copy genes and most (92.12%) of them showed no less than 10 types of copy number and also most (84.24%) genes did not show obvious population differentiation (*V*_ST_ ≤ 0.2, Additional file 4: Table S5). Except for the genes of unknown function, these multi-copy genes mainly (64.81%, Additional file 4: Table S5) consisted of coding genes of subunits of ribosomal proteins, ATP synthases, cytochromes, and some components of the transcription initiation complex and photosystems. These results confirmed that our approach could comprehensively detect CNVs with high sensitivity.

### The power evaluation of CtgRef-CNV pipeline

To validate the accuracy difference among CtgRef-CNV, CNVnator, and Delly, three copy number matrices of the 10 random CNV loci in the 15 accessions (Additional file 4: Table S6) were compared with the qPCR results (Additional file 4: Table S4) separately. According to the copy numbers verified by qPCR, we genotyped these 150 loci into DEL (deletion), DUP (duplication), and CN1 (normal type) to calculate the accuracy of each type of loci in the results of three software (Additional file 4: Table S6, see “Methods”). We found that the DUP accuracy of CNVnator was the highest (93.75%), while Delly had the highest accuracy in DEL detection (96.88%), and the accuracies of DUP (62.50%) and DEL (76.56%) of CtgRef-CNV were both between those of the other two software (Additional file 4: Table S6, and Table S7). In the detection of CN1 loci, there was little difference in the accuracies of the three software (Additional file 4: Table S6, and Table S7). Then, combining with the percentages of the three types of loci in these 15 accessions, we calculated the weighted accuracies of the three software (see “Methods”). And we found that our CtgRef-CNV had a higher weighted accuracy (88.93%) than Delly (86.26%) or CNVnator (84.84%, Additional file 4: Table S7), which indicates that the CtgRef-CNV is recommendable when using NGS data to call CNV.

We also assessed the difference of CNV results identified by CtgRef-CNV, CNVnator, and Delly. To analyze the overlap of high accuracy CNVs between three software, the filtered results of 15 accessions (Additional file 4: Table S8) by our rigid standards were selected as test data. The identification of the same CNV was referred to the method in the Wang et al. study [31]. We found that 10.16~18.97% (totaling 4939) of the CNVs generated by CNVnator, and 22.88~56.33% (totaling 7789) of CNVs generated by Delly, were overlapped with those identified by CtgRef-CNV (Additional file 4: Table S8). And the number of overlapped CNVs between CNVnator and Delly was much higher (22,462; Additional file 4: Table S8), which may be due to that these two algorithms were built on the results of reference-based read mapping. In addition, we also selected two CNV sets without preference from 15 accessions, and screened their accuracies by the IGV software [34]. The first set was the CNVs detected by CtgRef-CNV but not detected by CNVnator, among which 90.67% were also not detected by Delly, and the accuracy was 83.33% (Additional file 4: Table S9). The other set were the CNVs detected by CNVnator but not detected by CtgRef-CNV, among which 68% were also detected by Delly, and its accuracy was 86% (Additional file 4: Table S10). Moreover, the qPCR verification rate of integrative results was nearly 95% (mentioned above; Additional file 2: Figure S4 and Additional file 4: Table S4), which was much higher than that of each software (84.84%, 86.26%, or 88.93%; Additional file 4: Table S7). These results suggested that the integration of multiple algorithms is important for improving the CNV calling results based on NGS data. So, we used the integrative result of the three software as our final CNV set of each rice accession.

### The comparisons with published CNVs in rice

The accurate calling of CNVs is very important in genomics area, and many CNV data sets have been published in rice [31, 35–38]. In total, 641 CNVs were detected between Guangluai-4 and Nipponbare by CGH array [35]. We found that 302 out of their 641 CNVs were also detected by this study (Additional file 4: Table S11). Moreover, our results detected more than 7000 CNVs between Guangluai-4 and Nipponbare, which were not found in previous work [35]. These results indicated our method using NGS data could detect CNVs more comprehensively than the previously used array-based comparative genomic hybridization (CGH) technology. In 2017, a near complete reference genome of *indica* rice variety Shuhui-498 was assembled and 9909 presence variations (PVs, ≥ 500 bp) in the Nipponbare were identified, compared to the other 17 assembled rice genomes [36]. And 65.34% (6475) of these PVs was overlapped with 49.35% (6833/13,847) of our core DELs (Additional file 4: Table S12). There were 25,380 and 5813 genes identified as “Core” and “Dispensable” genes, respectively, in a pan-genome analysis (Additional file 2: Figure S6) [37]. Using the same criteria, most of the “Core” (23,941) genes were overlapped with the core genes identified in our work (Additional file 2: Figure S6). In addition, we extracted 9632 CNVs no shorter than 1 kb from the genomic variations of 3010 rice accessions [31] and found that 84.94% (8181) of them were overlapped with our core CNVs (Additional file 4: Table S13). Recently, the SVs of 3000 rice genomes were also analyzed by another group [38] and 183,943 CNVs (≥ 1 kb) were chosen from their SV results. By the method described in the Wang et al. [31], totally 52,883 core CNVs were generated. We found that 60.26% (31,865) of those core CNVs were overlapped with our core CNV set (Additional file 4: Table S14). All these results suggested that our CNV set is a valuable supplement to rice genome variation data set.

### The impact of CNVs on gene expression

One of the main effects of CNVs is to cause the alternation of gene expression levels [21, 23, 24, 39], by disrupting the gene, and affecting regulatory regions [10, 22, 25, 40, 41]. In this paper, considering the impact of short-read sequencing biases on the boundary identification of CNVs, only the genes, the coverage of which regions were more than 50% by CNVs, were used for correlation analysis. If a gene showed the same copy number in different accessions, the expression levels (TPMs) of the gene in the corresponding accessions would be grouped together. A copy number matrix of 14,435 genes in the 93 accessions was generated and only 2642 genes were selected for the correlation analysis between expression level and copy number by our strict standards (see “Methods”). A significantly positive correlation means that the expression level increases with the increase of copy number, while significantly negative correlation is that the expression level decreases with the increase of copy number (an adjusted *P* value < 0.05, see “Methods”).

**Fig. 2.**
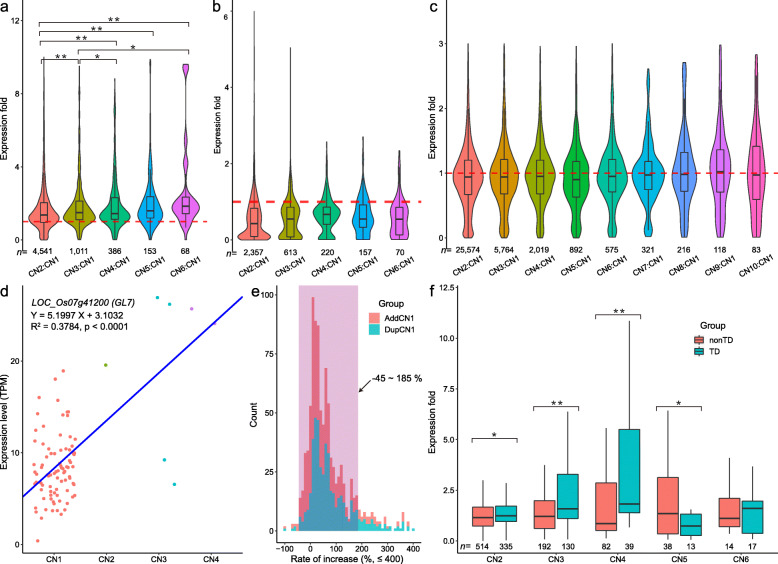
The impact of copy number variation on gene expression. **a–c** The distributions of expression folds (duplications to normal copy number) of the positively correlated genes (**a**), negatively correlated genes (**b**), and non-significantly correlated genes (**c**). CN1 means that its copy number is equal to 1 and so on. * and ** indicate significant difference at  *P* < 0.05 and *P* < 0.01, respectively, determined by the Tukey HSD test in R. The outliers (out of *μ* ± 3σ) are not displayed. **d** The correlation between copy number and expression level of the *GL7* (*LOC_Os07g41200*), and a TPM outlier from the CN1 group was discarded. The fitting and significance test of linear equation were performed by “trendline” function from the “basicTrendline” package in R. **e** The distributions of the increase rate of the two statistics of the positively correlated genes: AddCN1 (add one copy at a time) and DupCN1 (duplication compared to normal copy number). Values greater than 400% are not included in the figure. The data in the pink-shaded area accounted for more than 80% of each group. **f** The different effects of tandem duplications (TD) and non-tandem duplications (nonTD) on gene expression level. * and ** indicate significant difference at  *P*< 0.05 and *P* < 0.01, respectively, determined by the Wilcoxon test in R. The outliers (out of *μ* ± 3σ) are not displayed

Surprisingly, 82.32% of analyzed genes (Additional file 5: Table S15, and Additional file 6: Table S18) showed no significant correlations between the expression level and copy number and 13.17% of genes were significantly positive correlation (Additional file 5: Table S16 and Additional file 6: Table S18). Moreover, we found that 4.50% of genes showed negative correlation (Additional file 5: Table S17 and Additional file 6: Table S18). All the correlation results were further confirmed by the results of dosage effect analysis (Fig. 2a–c). For the *GL7* locus, a significant correlation was detected (Fig. 2d), which is consistent with previous work [23]. Analysis of variance revealed significant differences in expression levels among different copy numbers of approximately 75% of the correlated genes (*P* value < 0.05, Additional file 6: Table S18).

For positively correlated genes, the rate of increase in the expression level of duplicated genes mainly (account for > 81%) varied from −45% to 185% (Fig. 2e), by making a comparison between the expression level of genes and normal copy number (CN = 1). For each additional gene copy, the rate of the increase (comparing with CN1) in expression level also mainly (account for > 87%) varied from −45% to 185% (Fig. 2e) and this rate decreased as the copy number increased (Additional file 6: Table S19). While on the whole genome, the effect of duplication (not polyploidization) on gene expression was bidirectional, and the growth rate showed an approximately symmetric distribution on both sides of the vertical axis (Additional file 2: Figure S7), which reflected the robustness of gene expression regulation in vivo. In addition, we also found that the effect of tandem duplications on expression was generally stronger than that of dispersed ones, except when the copy number was 5 (Fig. 2f). These results indicated that the gene dosage effect is not exponential or linear, which is consistent with a previous report [42].

### The fates of duplicated genes

**Fig. 3.**
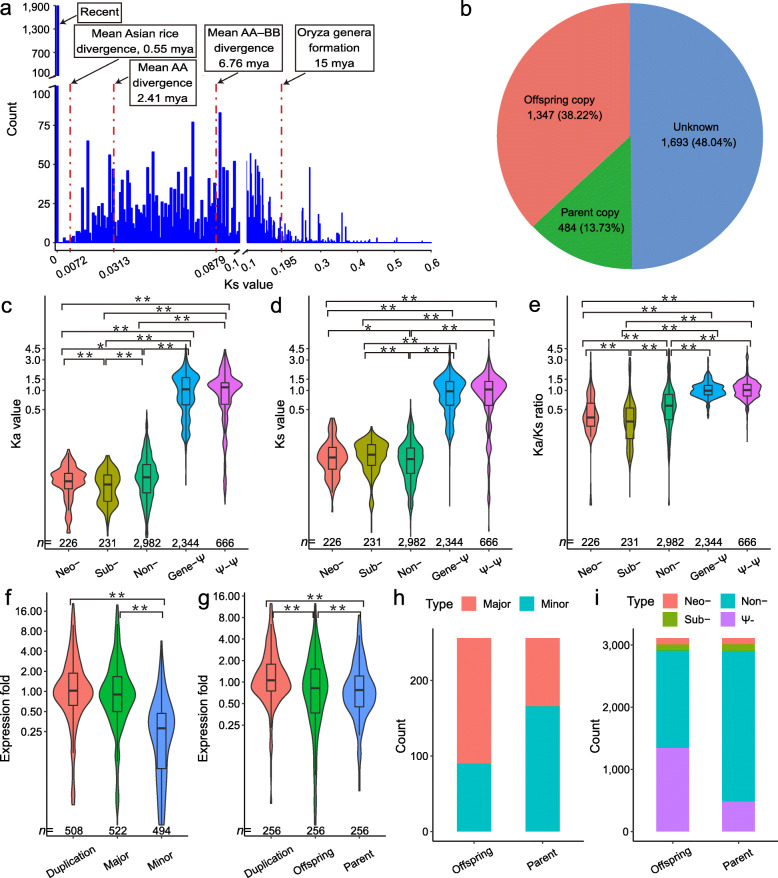
The expression and evolution of duplicated genes. **a** The *Ks* distribution of non-pseudogenetic duplicate pairs. The four *Ks* values (red dotted lines marked) represent key evolutionary events in the evolution of the *Oryza* genera, respectively, referring in the Stein et al. (2018). “Recent” means their *Ks* values are 0. **b** The component of the pseudogene copies. About half of the pseudogene copies were indistinguishable. And the rest was dominated by offspring copies. **c–e** The difference on *Ka* (**c**), *Ks* (**d**), *Ka*/*Ks* (**e**) and among neo-functionalized (Neo-), subfunctionalized (Sub-), undifferentiated (Non-) duplicated genes, functional gene-pseudogene pairs (Gene-Ψ), and pseudogene-pseudogene pairs (Ψ-Ψ). * and ** indicate significant difference at *P* < 0.05 and *P* < 0.01, respectively, determined by the Wilcoxon test in R. The outliers (out of *μ* ± 3*σ*) are not displayed. **f**, **g** The dosage sharing of major/minor (**f**) and parent/offspring (**g**) copies. The expression fold was normalized to the average TPM values of its corresponding normal gene (CN = 1). ** indicates a significant difference at *P* < 0.01 determined by the Tukey HSD test in R. The outliers (out of *μ* ± 3*σ*) are not displayed. **h**, **i** The proportions of major/minor copy (**h**) or differentiated copies (**i**) between parent and offspring copies

The main evolution consequences of duplicated genes are pseudogenization, neofunctionalization (Neo-), subfunctionalization (Sub-), and undifferentiating (Non-) [43–48]. The duplicate pairs, both can be assembled from NGS reads, were selected for further analysis. And in total, we identified 8163 gene pairs from assembled contigs of 93 accessions. By using the method of protein domain identification, we found that approximately 5.39% (440/8163) of duplicate pairs experienced the evolutionary divergence of gene function (Neo-/Sub-) and that approximately 36.46% (2976/8163) and 58.15% (4747/8163) of duplicate pairs experienced pseudogenization and undifferentiation, respectively (Additional file 6: Table S20). In our analysis, six stages (including the “Recent,” Fig. 3a) were used to determinate the age of gene duplication events, separated by “*Ks* (synonymous nucleotide substitution rate) > 0” and four another *Ks* values corresponding to the four speciation events during the divergence of the *Oryza* genera, according to previous study [49]. The *Ks* distribution of the non-pseudogenetic pairs (5338) implied that duplication events occurred throughout the six stages, especially in the recent past (more than 35%, Fig. 3a, Table 1). In each stage, the number of undifferentiating duplicate pairs was absolutely superior (more than 80%, except for 3/5 in the stage II, Table 1). Among the duplicate pairs of the stage I, eight duplicates subfunctionalized, indicating that functional differentiation can also be achieved in a short time. Interestingly, in the most recent two periods (stage I and II), all the functional differentiation pairs were subfunctionalization, but no neofunctionalization was observed. However, in other stages, the proportion of neofunctionalization pairs showed a growing trend with the aging of duplication (Table 1), confirming that subfunctionalization is an intermediate state of neofunctionalization [50].

**Table 1:** The statistics of non-pseudogenetic duplicate pairs at six stages

Stages	Range of *Ks*	Time (MYA)	Duplicate pairs^a^	Non-differentiated pairs	Differentiated pairs	Rate of differentiated pairs (%)	Neo-pairs	Subpairs	Rate of neo- in differentiated pairs (%)
I	= 0	Recent	1899	1891	8	0.42	0	8	0
II	0–0.0072	0–0.55	5	3	2	40	0	2	0
III	0.0072–0.0313	0.55–2.41	417	395	22	5.28	9	13	40.91
IV	0.0313–0.0879	2.41–6.76	1351	1181	170	12.58	103	67	60.59
V	0.0879–0.195	6.76–15	1369	1132	237	17.31	89	148	37.55
VI	> 0.195	> 15	297	271	26	8.75	25	1	96.15
Total	–	–	5338	4873	465	8.71	226	239	48.60

^a^A gene with a copy number of 3 will derive three duplicate pairs. And the genes with CDS length < 150 bp were excluded

To analysis the relationship between selective constraint and functional differentiation of duplicated genes, the *Ka* (nonsynonymous nucleotide substitution rate), *Ks*, and *Ka/Ks* ratio of different groups (Neo-, Sub-, Non-, Gene-Ψ, and Ψ-Ψ) were calculated using the Nei-Gojobori [51] method. The values of the duplicate pairs involved in pseudogene (Gene-Ψ, and Ψ-Ψ) were extremely higher than those of other duplicate pairs (all the *P* values were < 2.2 × 10^− 16^, Fig. 3c–e). 84.18% (2895/3439) of *Ka/Ks* values of functional duplicate pairs (Neo-, Sub-, and Non-) were less than 1, among which the undifferentiated pairs (Non-) were significantly higher than the differentiated pairs (Neo- and Sub-, *P* values: 7.292 × 10^− 9^ and < 2.2 × 10^− 16^, Fig. 3e), indicating that they were subject to different degrees of selection constraints, strengthened after functional differentiation. Compared with the neofunctionalizated pairs, the subfunctionalizated pairs accumulated significantly fewer nonsynonymous mutations (*P* value = 6.06 × 10^− 4^, Fig. 3c) in a slightly longer time (no significance, *P* value = 0.05929), so selection constraints on the subfunctionalizated pairs were significantly stronger (*P* value = 8.658 × 10^− 4^, Fig. 3e), and their sequences were more conservative.

### The asymmetric evolution of duplicated genes

Identifying the parent/offspring copies from gene pairs offers the opportunity to characterize the divergence of duplicated genes. A method based on conserved collinearity blocks in population (see “Methods”) was performed, and produced a total of 3129 distinguished duplicate pairs. The duplicated genes with more than two copies (18 groups) were discarded. Our results showed that the proportion of pseudogene copies in the offspring copy (43.30%) was much higher than that in the parent copy (15.56%, Fig. 3i). And correspondingly, in pseudogenes, the proportion of offspring copies (38.22%) was much higher than that of parent copies (13.73%, Fig. 3b). In addition, 77.92% (2424/3111) of the parent copies failed to change their function (Non- in the Fig. 3i). Thus, the parent copies mainly maintained the function of the original genes, especially when the offspring copies became pseudogenes, the percentage of the parent copies kept the original function intact was further increased (89.92%, Additional file 2: Figure S8). So, our results confirmed that the offspring copy is more likely to become pseudogene in rice and duplicated genes are asymmetrical in their evolutionary fates.

The copy-specific variations (CSVs), same to the singly unique nucleotides (SUNs) [52], were used to assign gene expression level. So the duplicate pairs without sequence divergence were excluded from our copy expression analysis. We totally obtained 548 duplicate pairs, whose copy-specific expression level could be split in the root RNA-Seq data from root tissue (Additional file 6: Table S21). Our results showed that the expression level of the major expressing copy was significantly higher than that of the minor one (Fig. 3f, *P* value < 1.0 × 10^− 7^), and most (97.57%) of these major-minor-expressed duplicate pairs could be traced back to the stages before the divergence of Asian rice (> 0.55 mya, *Ks* > 0.0072, Additional file 2: Figure S9). More interestingly, we found that the expression levels of offspring copies were also significantly higher expressed than that of the parent copies (Fig. 3g, *P* value = 4.44 × 10^− 5^). In the other way, the percentage of major copy in the offspring copies (64.84%) was higher than that in parent copies (35.16%, Fig. 3h). Hence, our results supported that the offspring copy is more likely to primarily express after the duplication event and duplicated genes are also asymmetrical in their expression levels. In conclusion, the asymmetric evolution of duplicated genes in rice is reflected in both the evolutionary fates and the expression levels.

## Discussion

Copy number variations reflect the evolution of genes and genomes, leading to local or population-specific adaptations to different environments and enriching population diversity. In recent years, several CNVs have been reported that they have contribution to the formation of the phenotypic diversity of important agronomic traits in rice, such as the duplication of *GL7* [23], deletions in *qSW5/GSE5* [53], duplication of the promoter of *IPA1* [25], and deletion of *sh1* [54]. However, all these studies focused on CNVs of individual locus. In order to uncover the relationship between CNVs and domestication on a genome-wide scale, we developed methods that could detect CNVs with high accuracy. Based on NGS data, the available methods for detecting CNVs have different disadvantages [7–9]. The read-pair (RP) methods are less effective in the low-complexity regions with repeat and are less accurate in detecting the actual copy number. The read depth (RD) methods fail to identify the precise breakpoints of CNVs. The split read (SR) methods rely on the read length and have low sensitivity in the low-complexity regions of the reference genome. The assembly-based (AS) methods consume a lot of computing resources and are difficult to accurately identify multiple copies with similar sequences. For CNV detection, a common strategy is to combine different methods. Here, we developed a pipeline integrating CNVnator (RD) [29], Delly (RP and SR) [30], and CtgRef-CNV (AS and RD), which add the advantages and avoid disadvantages of different methods. The shortcoming of our approach is that the filtering standards are strict and some real CNVs lacking reliable evidence (e.g., split reads, paired-end reads) to support may be discarded. Therefore, considering the high accuracy of results in rice, we believe that our method is powerful for the CNV analysis of other diploid species based on NGS data.

A genome-wide detection of CNVs had been performed previously in rice by CGH technology. They detected 641 CNVs between Guangliai-4 and Nipponbare [35]. Using deep NGS data, the first pan-genome of cultivated and wild rice was constructed, and a total of 10,872 presence-absence variations (PAVs) of genes in 67 accessions were also provided [37]. Then, in the 3000 Rice Genomes Project, 93,683 structural variations (SVs, containing 22,427 CNVs) and 48,098 gene PAVs were called among 453 high-coverage rice accessions [31]. In this study, we provided a high reliability CNVs set of 93 representative rice accessions, among which many genes had a gradient in copy number of the population. Therefore, our results may be beneficial to identify candidate genes regulating important agronomic traits. Our work enriches the understanding of the genetic basis of the formation and domestication of rice important phenotypes and provides insights into breeding of elite rice varieties.

Our large-scale analyses on the dosage effect of CNVs and gene expression in rice revealed that dosage effect was not exponential or linear and that the effect of tandem duplications on expression was generally stronger than that of sporadic ones. However, to our surprise 82.32% of analyzed genes (2175 genes) show no significant correlation between copy number and expression level and 4.5% of them (119 genes) showed negative correlation. There may be several possibilities for these results. First, copy number may not a dominant factor in affecting gene expression [10]. Second, instead of increasing their copy numbers, the alternative splicing of genes could be a more effective approach to adaption to abiotic stress in rice [55]. Third, the promoter or enhancer pairs of some duplicated genes may be differentiated, leading to differentiation of expression patterns [56–58], which can also weaken the effect of copy number on expression level. Moreover, the expressions of some genes are regulated by a negative feedback mechanism [59]; thus, the transcription levels could not always increase significantly with the increase of copy number. In addition, many *trans*-eQTLs (distance > 1 Mb, or on different chromosomes) were reported to be the predominant source of expression variation and contribute ~ 2-fold more to gene expression variance than local eQTLs [60–62]. So the *trans*-eQTL is also an important factor that influences expression level of genes, and our resequencing and transcriptomic data will provide great help for the identification of *trans*-eQTLs and local eQTLs in rice.

**Fig. 4.**
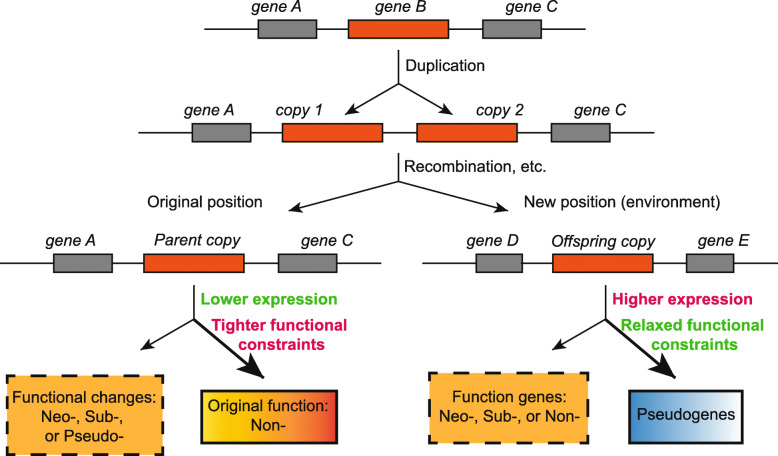
The model of asymmetric evolution of duplicated genes. This model is only applicable to cases where the two copies are separated after duplication. The copy that moved to a new genomic position is considered an offspring copy, and the other one is a parent copy. After moving to a new genomic region, the expression of offspring copy is no longer affected by the original regulation network and may be more active. At the same time, because of the functional redundancy, the functional constraint of the offspring copy is weaker than that of the original gene, so it is easier to accumulate more harmful mutations and degenerate into pseudogenes. On the contrary, due to the high expression of the new copy and feedback regulation, the expression level of the parent copy is relatively low. The parent copies are more limited because they were in their original positions, especially when the offspring copies accumulate more deleterious mutations that affect their functions, and the parent copies become more functionally constrained to maintain their original function. Thus, the evolution of two copies of a gene that were separated is asymmetrical in terms of expression and fate

Gene duplication is an important source of the origin of novel genes. It is widely believed that a novel gene is to become a pseudogene due to the accumulation of inactivating variations, but there are few large-scale analyses to reveal the fate of the two copies produced by gene duplication. Results from the *Drosophila* showed that neofunctionalization mainly occurred in the offspring copy, while the parent copy tended to retain the original function [47]. Our results support that parent copies tended to retain their original function, but the proportion of neofunctionalization in the parent copy or in the offspring copy showed no significant difference. More importantly, our results suggest that the offspring copies were more likely to be pseudogenized, thus exhibiting a different evolutionary fate from the parent copies. In addition, our work illustrates whether the evolution of duplicated genes is symmetrical or asymmetrical. Early works indicated that duplicated genes were found to evolve symmetrically, based on the comparison on the evolution rate in 39 organisms [63]. Latter, a wide range of cross-species or genome-wide evidence supported that the evolution of duplicated genes were asymmetrical. Their conclusions were based on the difference between two copies of duplicated genes on selection pressure [64], rate of evolution [65–71], and expression patterns [58, 65, 67, 68, 72–75]. Here, we detected the difference on the expression level and evolutionary fate between parent and offspring copies and proposed an asymmetric evolutionary model for the fate and expression of duplicated genes (Fig. 4). It is the offspring copy that tends to be more highly expressed and more likely to become pseudogene, which also reflects the difference in selection constraints between parent and offspring copies. The functional differentiation of duplicated genes provides an opportunity for the formation of new traits. Thus, to connect the evolved new functions of duplicated genes with certain traits in given landraces and cultivars would shed new light on the molecular design in crop breeding.

## Conclusion

Many important traits were reported to be affected by CNV. Herein, we developed a comprehensive pipeline and strict standards to call CNVs and provided a high accuracy CNV set for rice functional genomics research and molecular breeding application. Combined with transcriptome results, approximately 13% of the genes showed a significant correlation between their expression levels and copy numbers and gene dosage effect in rice was not exponential or linear. Based on the analyses on the evolutionary fates and expression levels of duplicated genes, we confirmed that maintenance of ancestral gene function and pseudogenization were the two main evolutionary fates and provided a new perspective for asymmetric evolution: the offspring copy mainly contributed to the expression levels and seemed more likely to become a pseudogene, whereas the parent copy tended to maintain the function of ancestral gene.

## Methods

### Plant growth, DNA, and RNA sequencing

The seeds of 93 rice accessions were grown according to Sun et al. [76], with some modifications: After germinating for 2 days at 28 °C, the seeds with consistent shoot were selected to place into bottom-cut PCR plate and grown for 7 days with nutrient solution varying from one quarter to half strength. The 7-day-old seedlings of uniform size and vigor were transplanted into holes in a cystosepiment placing over the top of the pots. The plants were grown for 3 weeks with half strength nutrient solution which was replaced with fresh solution every 3 days. The full chemical composition of the International Rice Research Institute (IRRI) nutrient solution contained 1.25 mM NH_4_NO_3_, 0.3 mM KH_2_PO_4_, 0.35 mM K_2_SO_4_, 1.0 mM CaCl_2_, 1.0 mM MgSO_4_·7H_2_O, 0.5 mM Na_2_SiO_3_, 20.0 μM Fe-EDTA, 9.0 μM MnCl_2_, 0.39 μM (NH_4_)_6_Mo_7_O_24_, 20.0 μM H_3_BO_3_, 0.77 μM ZnSO_4_, and 0.32 μM CuSO_4_ (pH 5.4–5.6). Plants were grown in a green house, with 70% relative humidity under fluorescence while light (150–200 μM m-2S-1) at 16-h light/8-h dark temperatures of 30/28 °C photoperiod.

For 93 rice accession genome resequencing, high-purity genomic DNA was extracted using QIAamp DNA Mini Kit (Qiagen). For genome resequencing, the DNA qualification, library construction, and resequencing was executed at Novogene (Beijing). High-throughput DNA Sequencing Pair-end sequencing was performed on Illumina® NovaSeq platform, with the read length of 150 bp at each end and average of 20 Gb sequencing data for each library.

The total RNA was extracted using TRIzol® Reagent (Invitrogen, Lot: 180702). All roots of the five seedlings of each accession were sampled for total RNA extraction. For rice transcriptome sequencing, the RNA qualification, library preparation, and RNA-Seq was executed at Berry Genomics Corporation (Beijing). After clustering of the index-coded samples performing on a cBot Cluster Generation System through Illumina Cluster Kit according to the manufacturer’s instruction, the library preparations were sequenced on an Illumina® Hiseq2500 platform and 125-bp paired-end reads were generated. The transcriptomic sequencing data of each accession was an average of 5 Gb.

### RNA-Seq data analysis

The Trimmomatic [77] package (version 0.32) was used to obtain clean reads. The adapters, Ns, and low-quality bases were removed, and the trimmed reads with a length less than 36 bp were also dropped. The Nipponbare RefSeq [28] (version 7.0) was used as the reference. All the clean reads were mapped to the reference using Tophat2 [78] (version 2.1.1) with default parameters. Next, reads with more than one reported alignments were excluded and TPM (transcripts per million) values were calculated with TPMCalculator [79] (version 0.0.3). Genes with TPM ≥ 0.5 were regarded as expressed genes.

### Resequencing reads filtering, mapping, and SNP calling and filtering

The Nipponbare RefSeq and its annotation were downloaded from MSU Rice Genome Annotation Project [28]. Reads containing adaptor sequences and low-quality reads were removed using cutadapt [80] (version 1.5) and SolexaQA [81] (version 3.1.3) according to the following criteria: (i) the Ns percent of one end > 5%, (ii) average quality score Q < 20, (iii) length < 75 bp after trimming. The filtered reads were mapped to the Nipponbare RefSeq (version 7.0) using BWA-MEM [82] (version 0.7.15-r1140). The mapped reads were sorted, and PCR duplicates were removed by SAMtools [83] (version 1.5). The mapped reads were also removed with a mapping quality < 30. Variants were called by SAMtools and BCFtools [83] (version 1.6). The raw SNPs were filtered using the following criteria: (i) the QUAL ≥ 100; (ii) raw read depth varies from 1000 to 50,000; (iii) MAF ≥ 5%; and (iv) biallelic sites.

### Copy number variations calling

Copy number variations were identified using our comprehensive pipeline (as previously mentioned), which consists of CNVnator [29] (version 0.3.4), Delly [30] (version 0.7.3; just for deletion calling), and CtgRef-CNV. Only CNVs with length no less than 1000 bp were selected to perform subsequent analyses.

For CNVnator, each accession was tested by several bin sizes from 100 to 1000 bp to make sure the ratio of average of RD (read depth) signal to standard deviation is between 4 and 5. For Delly package, only its DEL results were used in this project.

For the CtgRef-CNV pipeline, data was processed as follows:

(i) De novo assembly of each genome. The contigs were assembled using SOAPdenovo2 [84] package (version 2.04). The gaps in the draft assembly results were filled by the GapCloser [84] (version 1.12). The N50 length of the assemblies was evaluated after the small contigs of < 200 bp were excluded. The completeness of assembled genomes was evaluated by the BUSCO [85], using the “liliopsida_odb10” dataset as the reference.
(ii) Read mapping to its contigs. The clean reads were mapped to the contigs ≥ 1000 bp using the same package as mentioned above. The depth data was calculated through a non-overlapping sliding window method (the window size was 250 bp in our study) and corrected using the same method in the previous study [86].
(iii) Contig-reference alignment blocks. The contigs in (ii) were aligned with the Nipponbare RefSeq (version 7.0) by means of the nucmer program in the package MUMmer (version 3.23) [87] with the parameter “--maxmatch -c 90 -l 40.” The short alignment contig fragment contained in another one was filtered when its length rate (short/long) was less than 0.8. For two overlapping alignment contig fragments, (1) both were retained if the overlapping rate (overlap/longer) was no less than 0.7; otherwise, (2) the shorter alignment was removed if the length rate (short/long) was < 0.2.
(iv) Depth data of the reference. Based on the contig-reference alignment blocks, we transformed the contig depth into the reference depth result. If one contig was aligned to multiple regions in the reference, the mapped read depth of it would be subdivided equally to each aligned regions in the reference. On the other hand, for one region in the reference, its depth was accumulated by the subdivided depth from mapped contigs. Then the average depth of non-overlapping bins and chromosomes were calculated.
(v) CNV calling. The bins with CN_index (Average_depth_bin_ /Average_depth_Chr_) < 0.5 or ≥ 1.5 were selected for the candidates of deletion and duplication, respectively. The adjacent bins were linked to intervals if the CNV types were the same. Then the intervals were extended through the same approach as described in CNVnator algorithm. These extended intervals were candidated CNVs to be filtered.

### Filtering and integrating CNVs

The raw CNV results were filtered depending on calling method. For CNVnator CNV calls, regions with a copy number estimate between 0.8 and 1.4 were removed firstly [88]. A credible deletion should meet the following three conditions: (i) at least 5 discordant read pairs (with an insert size close to the size of the interval) or split reads supported within 500 bp upstream and downstream of the breakpoint; (ii) the coverage and (iii) the dp10_cvrg (coverage of sites with a depth ≥ 10) to be no more than 50%. For duplications, they should ensure as follows: (i) the coverage and (ii) the dp10_cvrg to be no less than 80%, and (iii) the dup_cvrg (coverage of sites with a CN_index ≥ 1.4) to be no less than 50%. For Delly (DEL) results, the copy number estimate should be less than 0.5, and other restrictions were the same as above. When filtering the results of CtgRef-CNV, the restrictions were a little stricter. The CNV calls with a copy number estimate between 0.4 and 1.7 were removed. In addition to the above conditions, the algn_cvrg (coverage of the alignment blocks) of a deletion should be < 50%, and ≥ 90% for a duplication. The filtered CNV calls were firstly merged by accession and integrated by the reference to the standard of same SVs reported by Wang et al. [31].

### The gene annotation of CNVs

The transposon genes are firstly filtered from the gff3 file in the MSU Rice Genome Annotation Project (release 7) [28]. And a candidate CNV gene was defined if no less than 50% of its gene body was covered by a CNV. In order to improve the accuracy of gene annotation, the gene with copy number of 0 was recalled to normal type (CN = 1) if the read coverage was ≥ 50% and the CN_index was ≥ 0.5. All the copy number matrices of accessions were merged into the population matrix.

### The qPCR verification of CNV results

The qPCR analysis was used to identify the relative copy number. Ten loci were randomly selected at 10 chromosomes among 15 accessions (Additional file 4: Table S5). The primers (Additional file 6: Table S22) were designed using Primer3 [89] (version 0.4.0) website. The *OsACTIN2* (*LOC_Os05g36280*) was used as inner reference gene. For each sample, the same amount of genomic DNA was used as template, according to the protocol of the ChamQ™ SYBR® Color qPCR Master Mix (Q441-02, Vazyme Biotech Co., Ltd., Nanjing, China), using the ABI StepOnePlus Real-Time PCR System, with three technical replicates. Amplification reactions were initiated with a denaturing step (95 °C for 10 min), followed by 40 cycles of denaturing (95 °C for 15 s), annealing, and extension (60 °C for 35 s). Data were analyzed by 2^−(∆∆Ct)^ method [90] to obtain relative copy number.

### The verification of the reported tandem duplication of *GL7* and the promoter of *IPA1*

The PCR analysis was performed using specific primers of duplication detection of *GL7* [23] and the promoter of *IPA1* [25] (Additional file 6: Table S22) on a T100™ Thermal Cycler (Bio-Rad) according to the manufacturer’s instructions. Briefly, in a 30 μL reaction system including 2 μL diluted DNA, 1.5 μL primers (10 μM/L), 4 μL dNTP (2.5 mM/L), 3 μL 10 × PCR buffer (Mg^2+^ plus), and 0.3 μL rTaq (5 U/μL, TaKaRa, R001B), amplification reactions were initiated with a denaturing step (98 °C for 2 min), followed by 30 cycles of denaturing (98 °C for 10 s), annealing (55 °C for 30 s), and extension (72 °C for 1 min). After PCR amplification, the products were detected by 1% agarose gel.

### The phylogenetic analysis and PCA

The filtered SNPs were used to calculate nucleotide diversity (π) and F-statistics (*F*_ST_), then the SNPs in the sliding windows with a *F*_ST_ ≤ 0.4 and a *π* no less than 0.1 × *π*_aver (the genome-wide average π) were used for phylogenetic analysis. The matrix of CNVs was used for genotyping by splitting duplications and deletions. If a duplication occurred in an accession, we assigned it to 1; otherwise, it was assigned to 0. It was the same for deletions. All the genotype values of CNVs were merged to construct the Neighbor-Joining tree by the APE package [91] (version 5.2) in R. The topological robustness was assessed by bootstrap analysis with 1000 replicates. For the PCA, CNVs with a copy number type > 3 or MAF < 0.03 were firstly removed. The copy number matrix was transformed into the plink format using our custom Perl script. Then the PCA was performed using the PLINK [92] (version 1.9).

### The calculation of weighted accuracy

In this study, we used the weighted accuracies to estimate the comprehensive accuracies of CNVnator, Delly, and CtgRef-CNV. And it was determined as follows: *R*_*w*_*=* ∑*P*_*t*_ ∗ *R*_*t*_, where *P*_*t*_ and *R*_*t*_ are the percentages and accuracies of the DUP (CN ≥ 2), DEL (CN = 0), and CN1 types, respectively. The percentage of each type of loci was computed based on the copy number matrix of the 15 accessions. And the accuracy depended on the called and qPCR results. For the DEL, and CN1 loci, only if the CN_call_ (copy number called by software) of a locus in an accession was equal to the CN_qPCR_ (copy number verified by qPCR), we treated it correct. And for the DUP locus, if the CN_call_ was 0, or 1, we said it was completely wrong; however, if the CN_call_ was no less than 2, we would treat it partially (not equal to the CN_qPCR_) or completely (equal to the CN_qPCR_) correct and the number of correct DUPs would be accumulated by CN_Min_/CN_Maj_, where the CN_Min_ and CN_Maj_ were minor and major values of the array: [CN_call_, CN_qPCR_], respectively. In particular, if the CN_call_ of a DUP locus was the same as its CN_qPCR_, the number of correct DUPs would also be accumulated by 1.

### The correlation analysis

The CNVs were annotated genes sample by sample, and then all the gene copy number matrix were integrated to the total matrix of population. Only the genes impacted by CNV (minimum 50% gene model overlap) and with a change fold (max/min) no less than 1.1 were selected to carry out the correlation analysis between copy number and gene expression level. The TPM outliers (out of the range of *μ* ± 3σ) and its copy number were filtered. Then the copy number less than three replicates was removed. If the remaining copy number was of only two types, and the smaller copy number was equal to 0, these genes were also discarded before the correlation analysis. To minimize the impact of population structure, the copy numbers of genes filtered by the above standards were used to calculate the *V*_ST_ values [4] and only the genes with *V*_ST_ values no more than 0.4 were performed for the next analyses. A significance level of 0.05 was set for the corrected *t*-test *P* values (Benjamini–Hochberg method [93]).

### The copy-specific expression

The duplicate pairs, of which the copy number is in agreement with the number of duplicates in the assembly contigs, were selected. The pseudogenes and the coding sequences (CDSs) were predicted using GeneWise [94] (version 2.4.1). Then the paired CDSs were aligned by ClustalW2 [95] (version 2.0.12) to identify copy-specific variations (CSVs), similar to the method described [52]. The CSVs were used to count copy-specific reads in the RNA-Seq data to calculate the expression level of each copy. Within each pair, we classified the copy with higher expression level as “major” copy and its partner as the “minor” copy. The expression difference between “major” copy and “minor” copy was defined as the expression difference of the corresponding duplicate pair. If the expression level of the major copy was more than two times as that of minor one, we said that there was a phenomenon of copy dominance expression in this pair.

### The *K*a, *K*s, and the divergence time calculations

The CDSs of the non-pseudogenic duplicate pairs used in copy-specific expression analysis were translated into proteins. The number of nonsynonymous substitutions per nonsynonymous site (*Ka*) and the number of synonymous substitutions per synonymous site (*Ks*) were calculated by the yn00 program in the PAML4 packages [96] using the Nei-Gojobori [51] method. The alignment involved in pseudogene was performed by MASCE [97] (version 2.03) software. The time since divergence (*T*) of duplicates was calculated as *T* = *Ks*/2*μ*, where *μ* corresponds to the absolute substitution per synonymous site per year, and here, we use substitution rate of the grass *Adh* sequences (6.5 × 10^− 9^) [98].

### The prediction of the parent and offspring copies

Based on the alignment position of each duplicate pair in the Nipponbare genome, four adjacent genes (two upstream and two downstream) were aligned with the assembled genomes of accessions with the normal copy number. Given the short length of assembled contigs using the NGS reads, no less than two adjacent genes were assembled into the same contig with the target gene which was the effective evidence that it was the same colinearity near the target gene between the accession and the Nipponbare. If there were more than 10 accessions (or most of the accessions) supporting the gene position in the Nipponbare, the colinearity was considered to be conserved. Then the same criterion was enforced in all the alignment results. The copy in the colinearity block was identified as the parent copy and the others were the offspring copies. However, for tandem duplicate pairs, it might be impossible to distinguish which one was the parental copy and which was the offspring.

### The identification of neofunctionalization and subfunctionalization

All the protein sequences of the non-pseudogenic duplicate pairs and the corresponding references were used for the domain analysis through the InterProScan [99] (version 5.21-60.0), with an E-value no more than 1.0 × 10^− 5^. A copy contained fewer domains than its reference counterpart which meant there was a subfunctionalization event, whereas a copy containing more or new domains indicated a neofunctionalization event. If the duplicate pair had the same domains, it was not possible to determine whether there was neofunctionalization or subfunctionalization event without the evidence of expression patterns.

## Supplementary information

**Additional file 1: Table S1.** The summary of 93 representative rice accessions for whole genome re-sequencing, de novo assembling and RNA-Seq.

**Additional file 2.** Supplementary Table legends and supplementary Figures.

**Additional file 3: Table S2.** The copy number matrix of 93 rice accessions.

**Additional file 4: Table S3.** The tandem duplications identified around GL7 loci and the promotor of IPA1. a equal to Average_depthCNV/Average_depthChr. b the read with a insert size close to the length of the CNV. c the region within 500 bp upstream and downstream of the breakpoint were used to detect the discordant reads. **Table S4.** The copy number matrix of the 10 CNVs identified by qPCT. **Table S5.** Genes with high copy number (≥10) in more than 5 accessions. **Table S6.** The copy number matrices of 10 loci in 15 accessions called by CNVnator, Delly, and CtgRef-CNV. The DUP, DEL, and CN1 loci, were marked as yellow, purple, and green, respectively. **Table S7.** The weighted accuracies of three softwares based on the qPCT results of 10 loci in 15 accessions. **Table S8.** The comparisons of CNV results generated by three methods. **Table S9.** The accuracy of the selected 150 CNVs detected by CtgRef-CNV but not detected by CNVnator. **Table S10.** The accuracy of the selected 150 CNVs detected by CNVnator but not detected by CtgRef-CNV. **Table S11.** The comparison of CNVs between Yu et al. and present study. **Table S12.** The comparison of CNVs between Du et al. and present study. **Table S13.** The comparison of CNVs between Wang et al. (3 K rice genomes) and present study. **Table S14.** The comparison of CNVs between Fuentes et al. (3 K rice genomes) and present study.

**Additional file 5: Table S15.** The loci without significant correlation between copy number and expression level. **Table S16.** The positively correlated loci between copy number and expression level. **Table S17.** The negatively correlated loci between copy number and expression level.

**Additional file 6: Table S18.** The statistics of correlation analysis. a for each gene, the TPM values were divided into groups by different copy numbers. Difference analysis were performed in R. If there was/were significant difference between at least two groups of TPM values, this gene was defined as Exp_diff gene. **Table S19.** The changes in the average rate of increase with the copy number gradient of positively correlated genes. Values are means ± s.d.. **Table S20.** The fate of duplicated genes. a corresponds to the pairs at least one copy pseudogenized. b-d correspond to the pairs at least one copy neo-functionalized, sub-functionalized, and neo-functionalized/sub-functionalized, respectively. e corresponds to the pairs whose copy numbers were in line with the assembly results of 93 rice accessions. **Table S21.** The number of copy specific reads. **Table S22.** Primers used in this study.

## Data Availability

The raw resequencing data of the 93 accessions have been deposited to the SRA at the NCBI under the BioProject IDs PRJNA522896 [100] and PRJNA535372. And for the raw transcriptome data, the BioProject ID at the NCBI was PRJNA539946. All the SRA accessions are listed in the Additional file 1: Table S1. The scripts used in the CtgRef-CNV pipeline are available under the link: https://github.com/flzh628/CtgRef-CNV.
