# Supplementary material for: A genome-wide survey of copy number variations reveals an asymmetric evolution of duplicated genes in rice
Source: BMC Biol. 2020 Jun 26;18:73. doi: 10.1186/s12915-020-00798-0 (PMC7318451; doi:10.1186/s12915-020-00798-0)
Supplement: Supplementary file 2 — Additional file 2. Supplementary Table legends and supplementary Figures. [file 12915_2020_798_MOESM2_ESM.docx]

Additional file 2

## **A genome-wide survey of copy number variations reveals an asymmetric evolution of duplicated genes in rice**

Fengli Zhao^1,5^, Yuexing Wang^2,5^, Jianshu Zheng^1,5^, Yanling Wen^3,5^, Minghao Qu^1^, Shujing Kang^1^, Shigang Wu^1^, Xiaojuan Deng^1^, Kai Hong^1^, Sanfeng Li^2^, Xing Qin^1^, Zhichao Wu^1^, Xiaobo Wang^1^, Cheng Ai^1^, Alun Li^1^, Longjun Zeng^1,4^, Jiang Hu^2^, Dali Zeng^2^, Lianguang Shang^1^, Quan Wang^1^, Qian Qian^1,2^, Jue Ruan^1*^ and Guosheng Xiong^1,4*^

# Table of contents

[**A genome-wide survey of copy number variations reveals an asymmetric evolution of duplicated genes in rice** 1](#_Toc36389407)

[Table of contents 2](#_Toc36389408)

[Supplementary Table legends 4](#_Toc36389409)

[Table S1. The summary of 93 representative rice accessions for whole genome re-sequencing, *de novo* assembling and RNA-Seq. 4](#_Toc36389410)

[Table S2. The copy number matrix of 93 rice accessions. 4](#_Toc36389411)

[Table S3. The tandem duplications identified around *GL7* loci and the promotor of *IPA1*. 4](#_Toc36389412)

[Table S4. The copy number matrix of the 10 CNVs identified by qPCT. 4](#_Toc36389413)

[Table S5. Genes with high copy number (≥10) in more than 5 accessions. 4](#_Toc36389414)

[Table S6. The copy number matrices of 10 loci in 15 accessions called by CNVnator, Delly, and CtgRef-CNV. 4](#_Toc36389415)

[Table S7. The weighted accuracies of three softwares based on the qPCT results of 10 loci in 15 accessions. 4](#_Toc36389416)

[Table S8. The comparisons of CNV results generated by three methods. 4](#_Toc36389417)

[Table S9. The accuracy of the selected 150 CNVs detected by CtgRef-CNV but not detected by CNVnator. 5](#_Toc36389418)

[Table S10. The accuracy of the selected 150 CNVs detected by CNVnator but not detected by CtgRef-CNV. 5](#_Toc36389419)

[Table S11. The comparison of CNVs between Yu et al. and present study. 5](#_Toc36389420)

[Table S12. The comparison of CNVs between Du et al. and present study. 5](#_Toc36389421)

[Table S13. The comparison of CNVs between Wang et al. (3K rice genomes) and present study. 5](#_Toc36389422)

[Table S14. The comparison of CNVs between Fuentes et al. (3K rice genomes) and present study. 5](#_Toc36389423)

[Table S15. The loci without significant correlation between copy number and expression level. 5](#_Toc36389424)

[Table S16. The positively correlated loci between copy number and expression level. 5](#_Toc36389425)

[Table S17. The negatively correlated loci between copy number and expression level. 5](#_Toc36389426)

[Table S18. The statistics of correlation analysis. 5](#_Toc36389427)

[Table S19. The changes in the average rate of increase with the copy number gradient of positively correlated genes. 6](#_Toc36389428)

[Table S20. The fate of duplicated genes. 6](#_Toc36389429)

[Table S21. The number of copy specific reads. 6](#_Toc36389430)

[Table S22. Primers used in this study. 6](#_Toc36389431)

[Supplementary Figures 7](#_Toc36389432)

[Figure S1. The comprehensive pipeline of CNV calling. 7](#_Toc36389433)

[Figure S2. The CNV numbers of 500-kb non-overlap windows at 12 chromosomes. 8](#_Toc36389434)

[Figure S3. The statistics of CNV calling for the 93 rice accessions. 9](#_Toc36389435)

[Figure S4. The qPCR verification of 10 CNV locus using 15 rice accessions. 10](#_Toc36389436)

[Figure S5. The phylogenetic tree and PCA analysis of the 93 rice accessions. 11](#_Toc36389437)

[Figure S6. The venn diagram of pan-genome results between Zhao et al. (2018) and our CNV data. 12](#_Toc36389438)

[Figure S7. The impact of duplication on gene expression. 13](#_Toc36389439)

[Figure S8. The function component of the parent copies when its offspring copies were pseudogenes. 14](#_Toc36389440)

[Figure S9. The Ks distribution of major-minor duplicated pairs. 15](#_Toc36389441)

# Supplementary Table legends

#### **Table S1. The summary of 93 representative rice accessions for whole genome re-sequencing, *de novo* assembling and RNA-Seq.**

#### **Table S2. The copy number matrix of 93 rice accessions.**

#### **Table S3. The tandem duplications identified around *GL7* loci and the promotor of *IPA1*.**

^a^ equal to Average_depth_CNV_/Average_depth_Chr_.

^b^ the read with a insert size close to the length of the CNV.

^c^ the region within 500 bp upstream and downstream of the breakpoint were used to detect the discordant reads.

#### **Table S4. The copy number matrix of the 10 CNVs identified by qPCT.**

#### **Table S5. Genes with high copy number (≥10) in more than 5 accessions.**

#### **Table S6. The copy number matrices of 10 loci in 15 accessions called by CNVnator, Delly, and CtgRef-CNV.**

The DUP, DEL, and CN1 loci, were marked as yellow, purple, and green, respectively.

#### **Table S7. The weighted accuracies of three softwares based on the qPCT results of 10 loci in 15 accessions.**

#### **Table S8. The comparisons of CNV results generated by three methods.**

#### **Table S9. The accuracy of the selected 150 CNVs detected by CtgRef-CNV but not detected by CNVnator.**

#### **Table S10. The accuracy of the selected 150 CNVs detected by CNVnator but not detected by CtgRef-CNV.**

#### **Table S11. The comparison of CNVs between Yu et al. and present study.**

#### **Table S12. The comparison of CNVs between Du et al. and present study.**

#### **Table S13. The comparison of CNVs between Wang et al. (3K rice genomes) and present study.**

#### **Table S14. The comparison of CNVs between Fuentes et al. (3K rice genomes) and present study.**

#### **Table S15. The loci without significant correlation between copy number and expression level.**

#### **Table S16. The positively correlated loci between copy number and expression level.**

#### **Table S17. The negatively correlated loci between copy number and expression level.**

#### **Table S18. The statistics of correlation analysis.**

a for each gene, the TPM values were divided into groups by different copy numbers. Difference analysis were performed in R. If there was/were significant difference between at least two groups of TPM values, this gene was defined as Exp_diff gene.

#### **Table S19. The changes in the average rate of increase with the copy number gradient of positively correlated genes.**

Values are means ± s.d..

#### **Table S20. The fate of duplicated genes.**

a corresponds to the pairs at least one copy pseudogenized.

b-d correspond to the pairs at least one copy neo-functionalized, sub-functionalized, and neo-functionalized/sub-functionalized, respectively.

e corresponds to the pairs whose copy numbers were in line with the assembly results of 93 rice accessions.

#### **Table S21. The number of copy specific reads.**

#### **Table S22. Primers used in this study.**

# Supplementary Figures

**
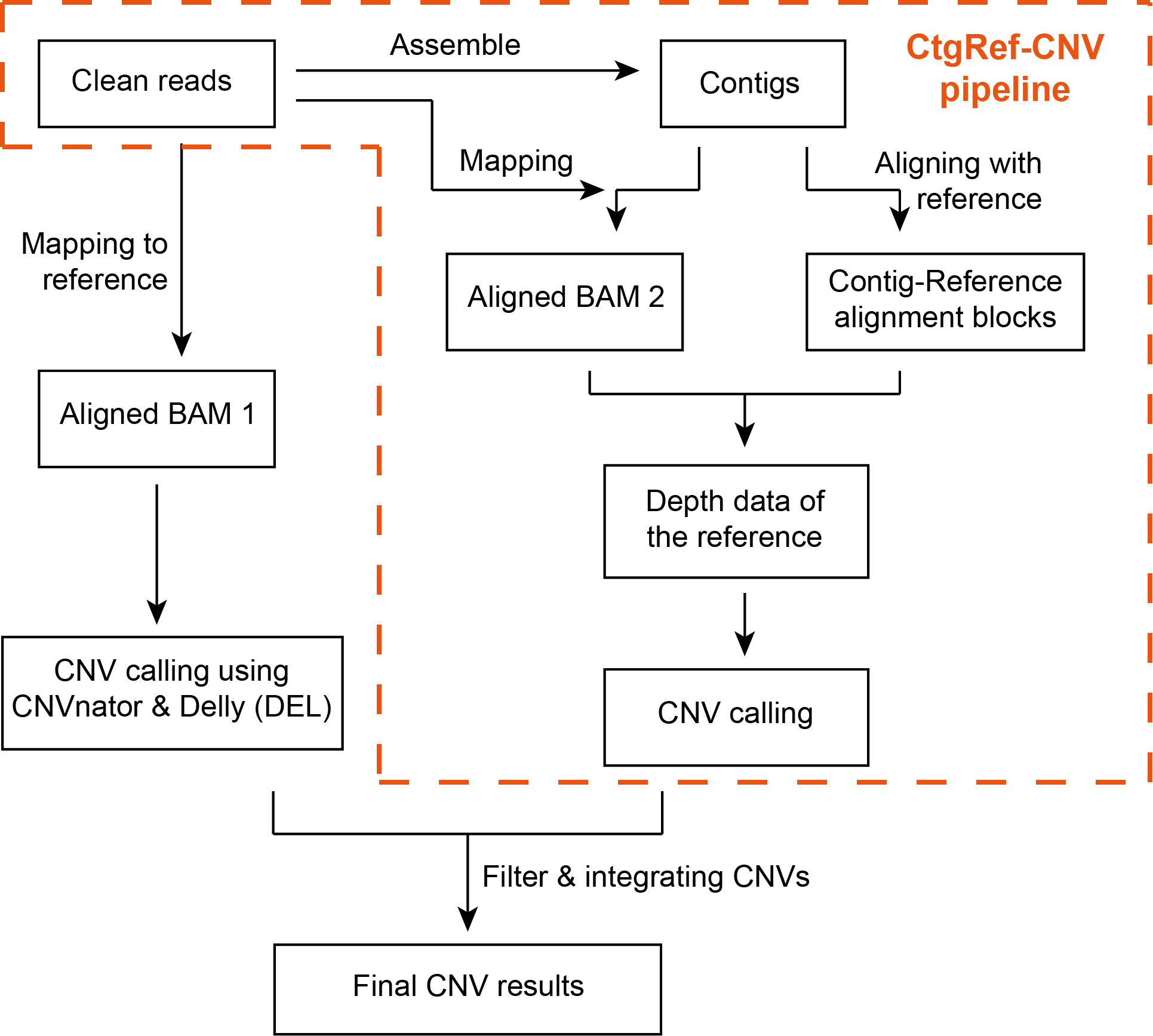
**

#### **Figure S1. The comprehensive pipeline of CNV calling.**

Steps in the red dotted box were the algorithm named “CtgRef-CNV”. This pipeline combines read depth and de novo assembly methods. To reduce the false positive rate, a strict standard were proposed to filter the CNV results. At last, the CNVs of each accession were integrated.

**
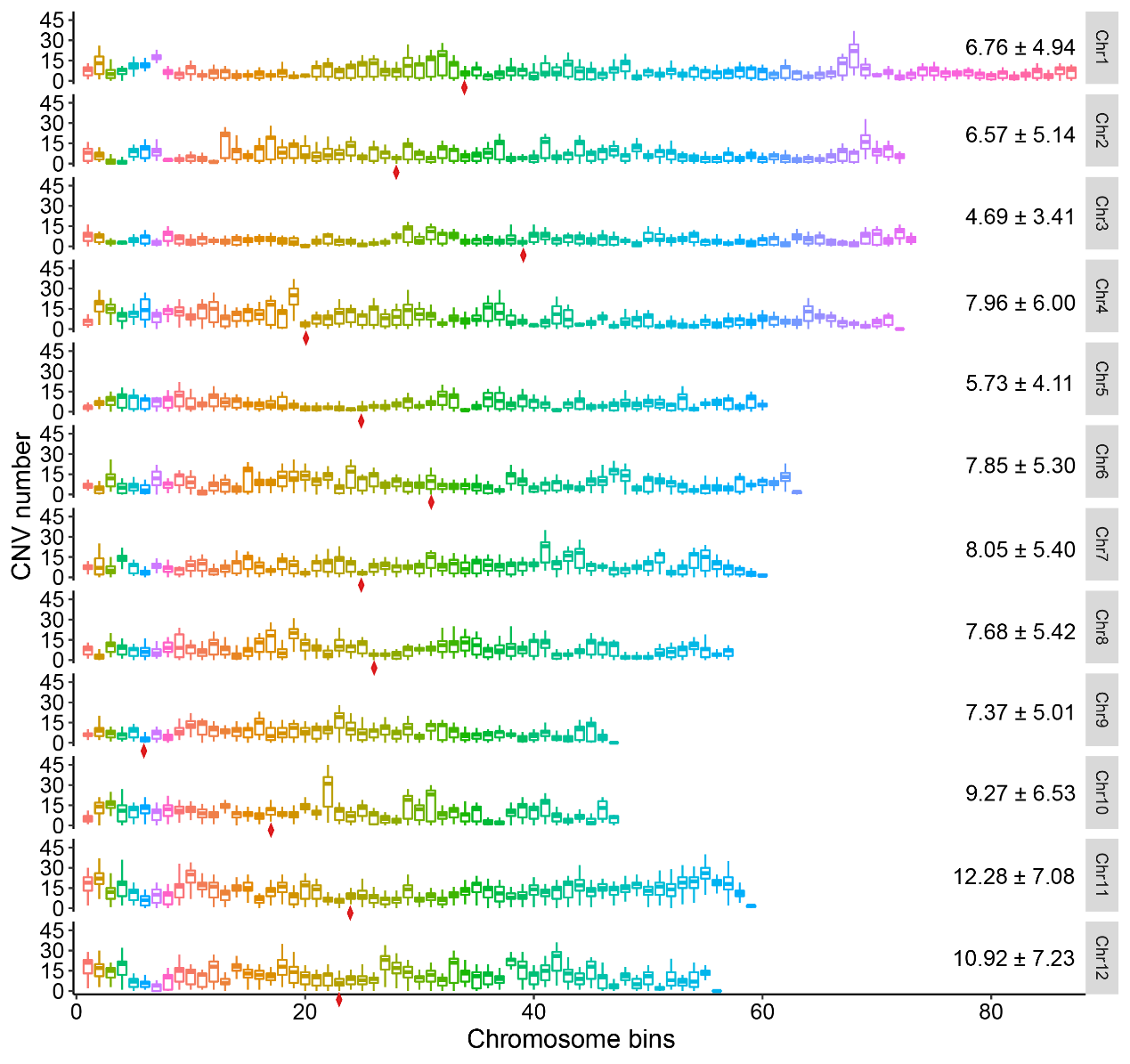
**

#### **Figure S2. The CNV numbers of 500-kb non-overlap windows at 12 chromosomes.**

The red diamonds marked the positions of the centromere. Each boxplot represents the CNV number of the corresponding location in the 93 rice accessions.

**
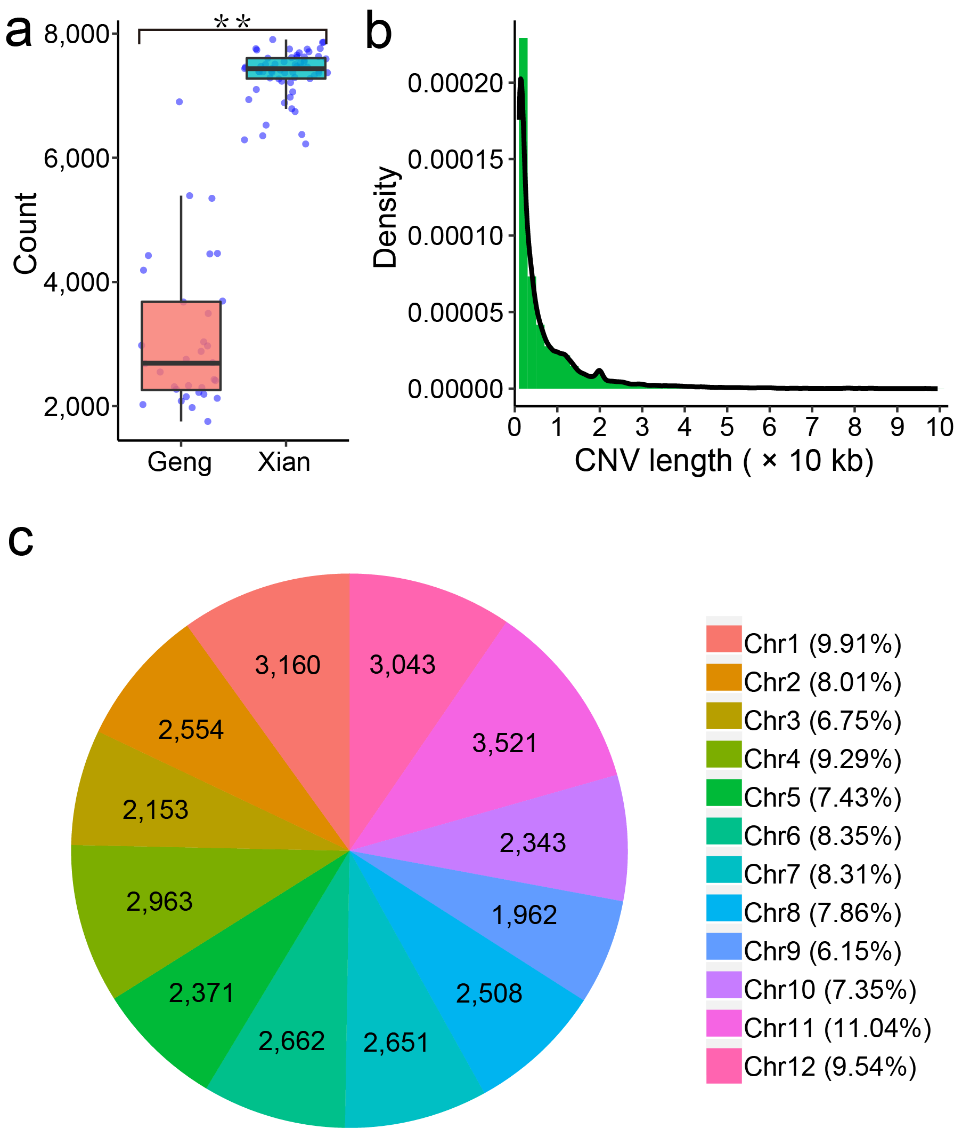
**

#### **Figure S3. The statistics of CNV calling for the 93 rice accessions.**

a, The difference on the CNV number between *O. sativa* Xian group and *O. sativa* Geng Group, compared with the Nipponbare RefSeq. ** indicate a significant difference at *P* < 0.01 determined by t-test in R.

b, The length distribution of population-integrated CNVs (shorter than 100 kb).

c, The Chromosome distribution of population-integrated CNVs.

**
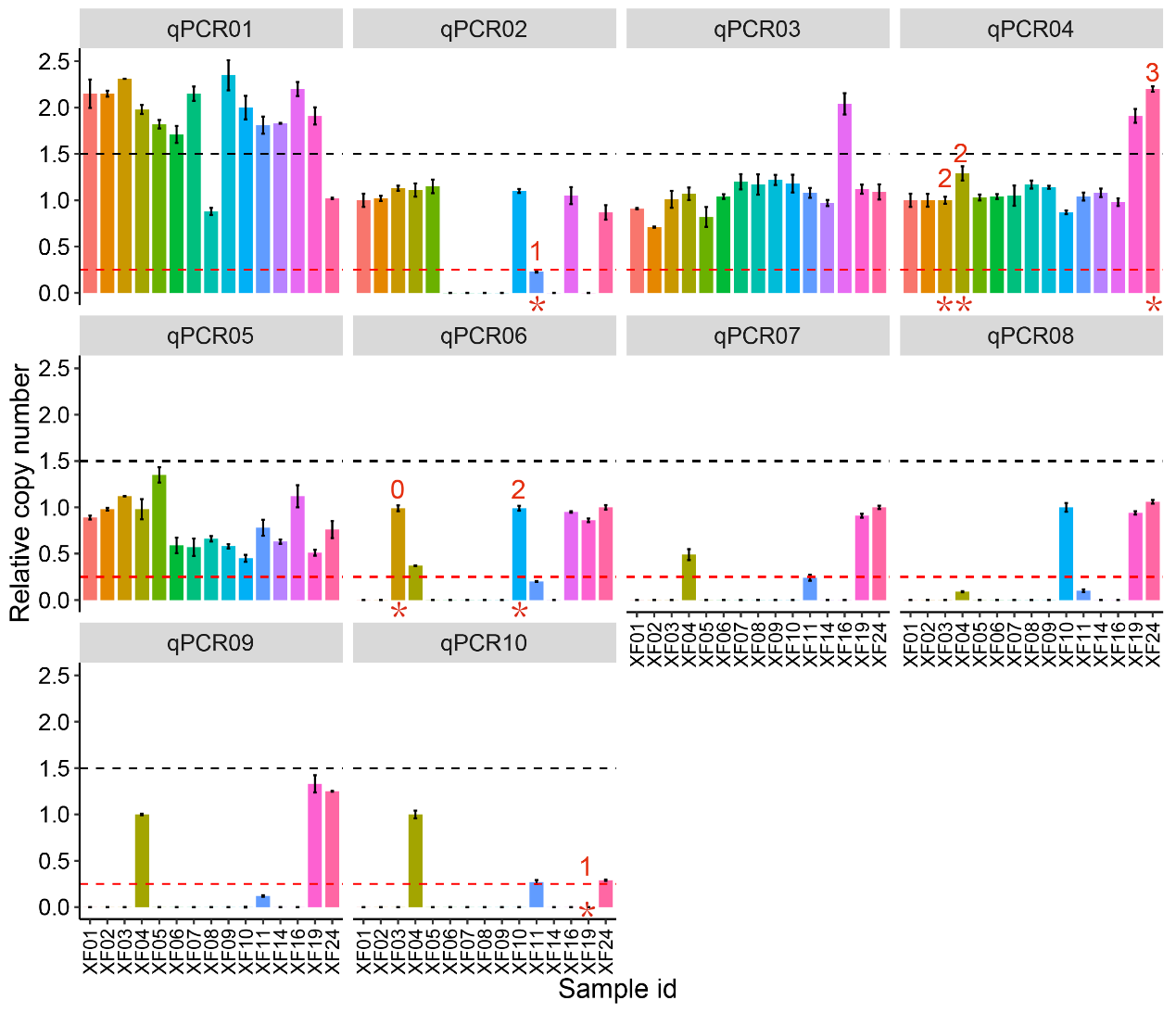
**

#### **Figure S4. The qPCR verification of 10 CNV locus using 15 rice accessions.**

If the relative copy number is less than 0.25, it is more likely to be consistent with deletion, on the contrary, a reliable duplication should have a relative copy number no less than 1.5. The red and black dotted lines indicate the thresholds of relative copy number for deletion and duplication, respectively. The results where the CNV calling were inconsistent with the verification were marked by red asterisks and the the CNV calling results were marked above the column. So, the accuracy rate was nearly (150 – 7)/150 = 95.33%.

**
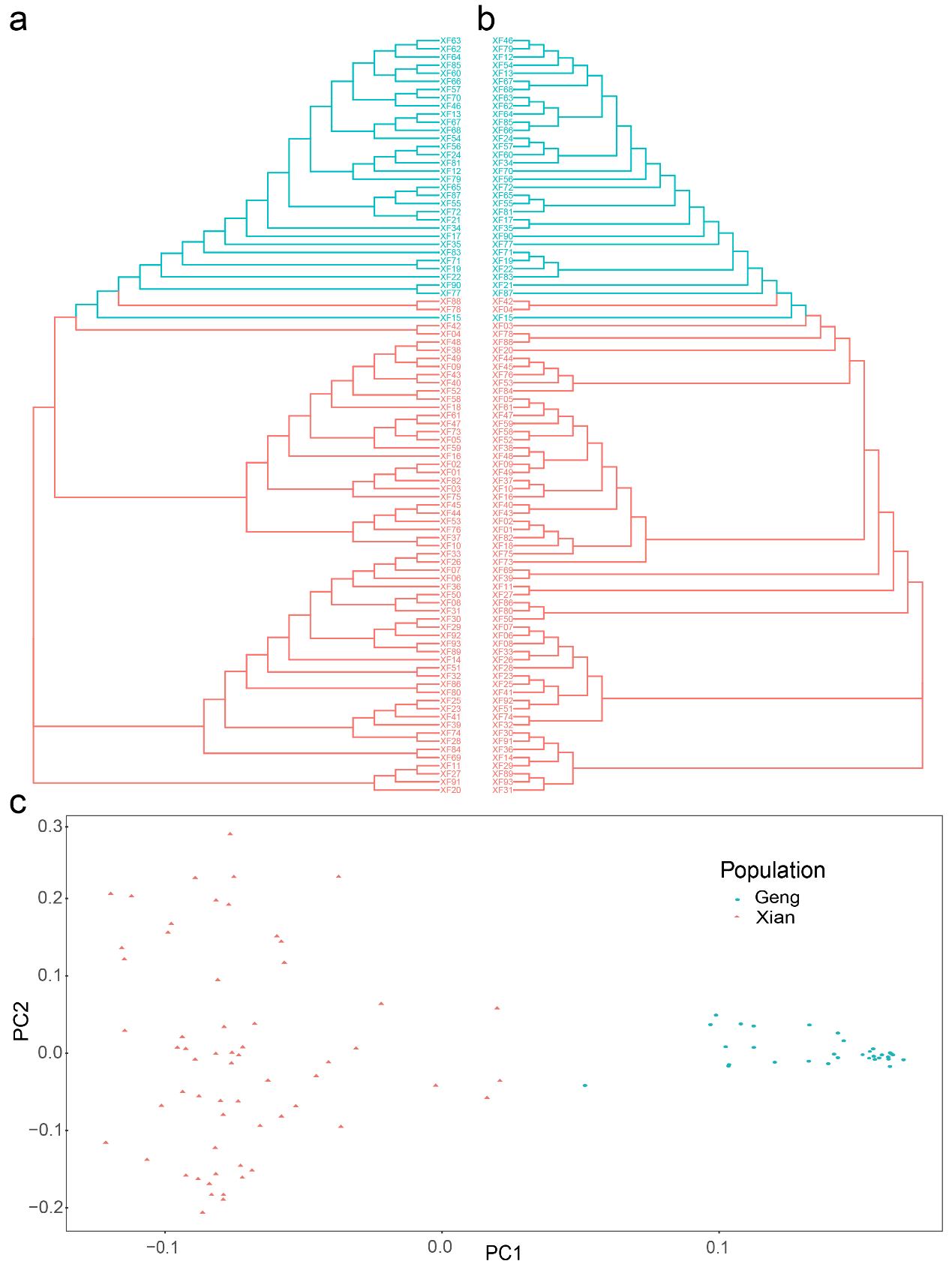
**

#### **Figure S5. The phylogenetic tree and PCA analysis of the 93 rice accessions.**

a-b. The phylogenetic tree analysis based on SNPs and CNVs. The *O. sativa* Xian accessions and *O. sativa* Geng accessions were marked in red and blue, respectively.

c. The PCA result based on CNVs. Two subspecies groups were clearly separated.


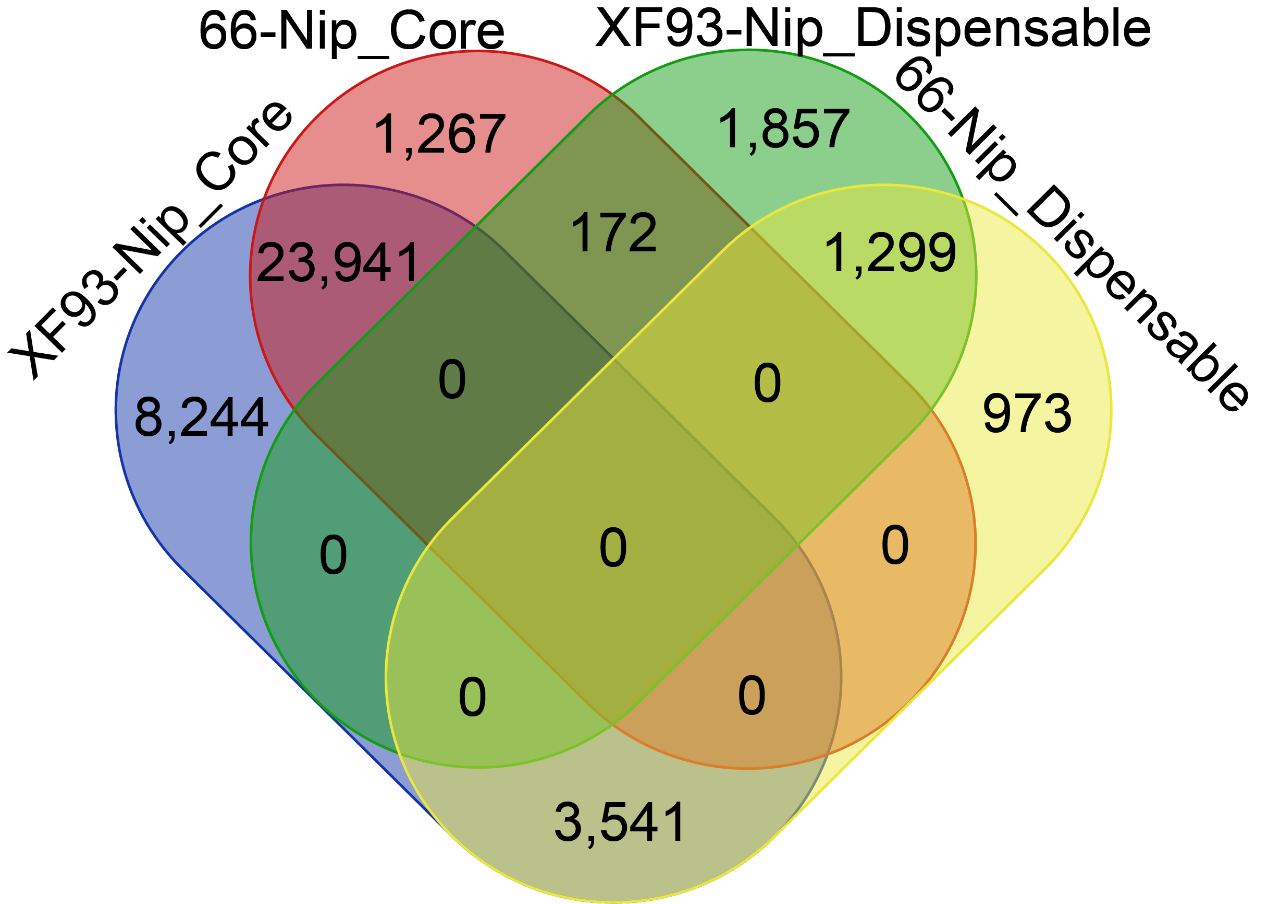


#### **Figure S6. The venn diagram of pan-genome results between Zhao et al. (2018) and our CNV data.**

**
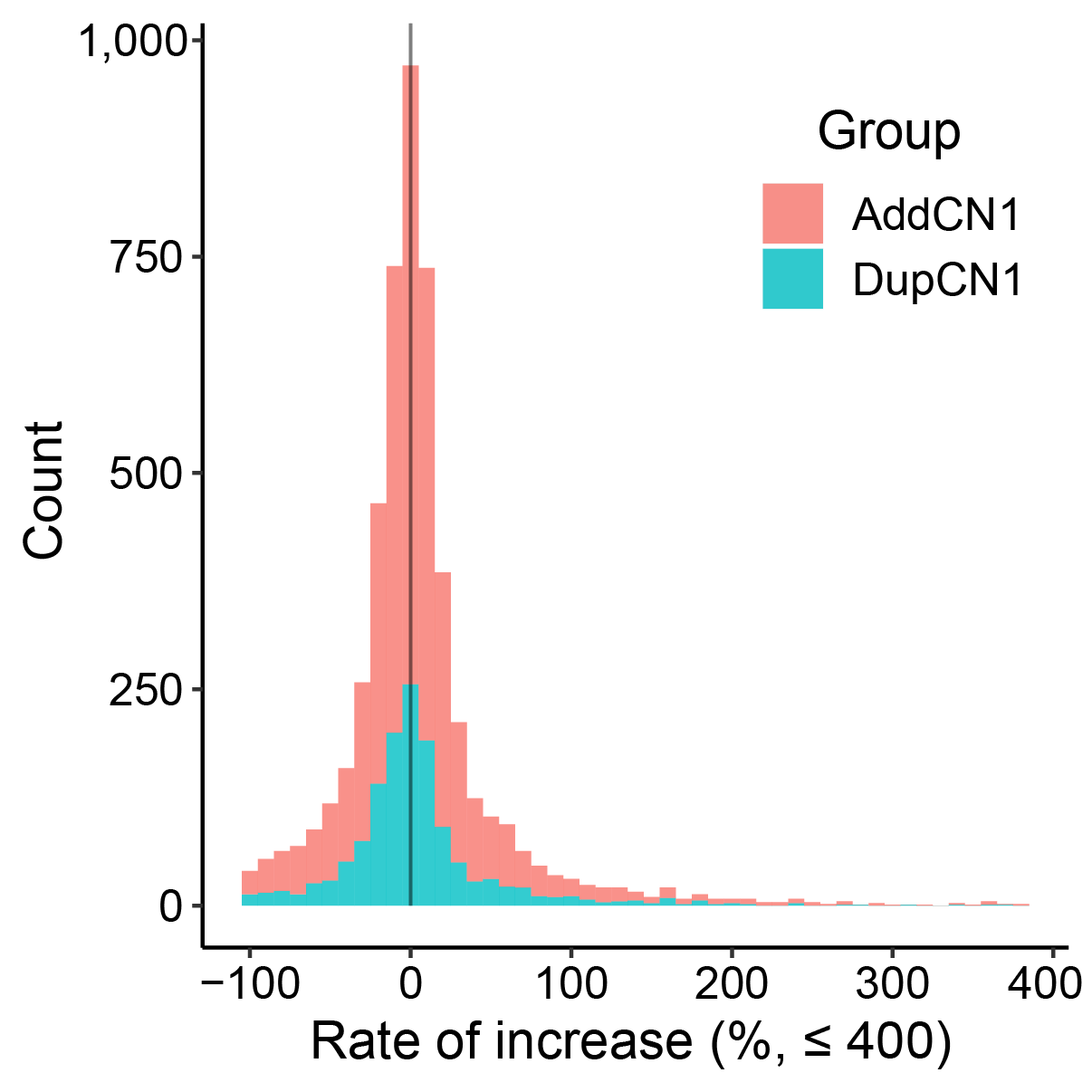
**

#### **Figure S7. The impact of duplication on gene expression.**

The distributions of the increase rate of the two statistics of genes involved in correlation analysis: AddCN1 (add one copy at a time) and DupCN1 (duplication compared to normal copy number). Values greater than 400% are not included in the figure.

**
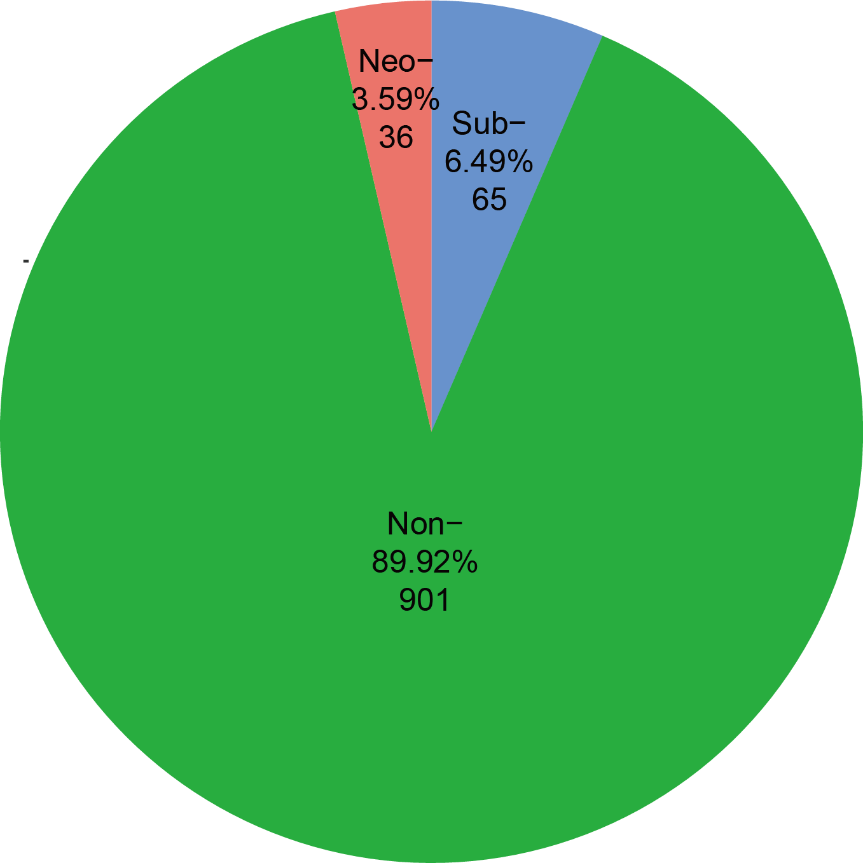
**

#### **Figure S8. The function component of the parent copies when its offspring copies were pseudogenes.**

Non-, Neo- and Sub- represent undifferentiation, neofunctionalization and subfunctionalization, respectively.

**
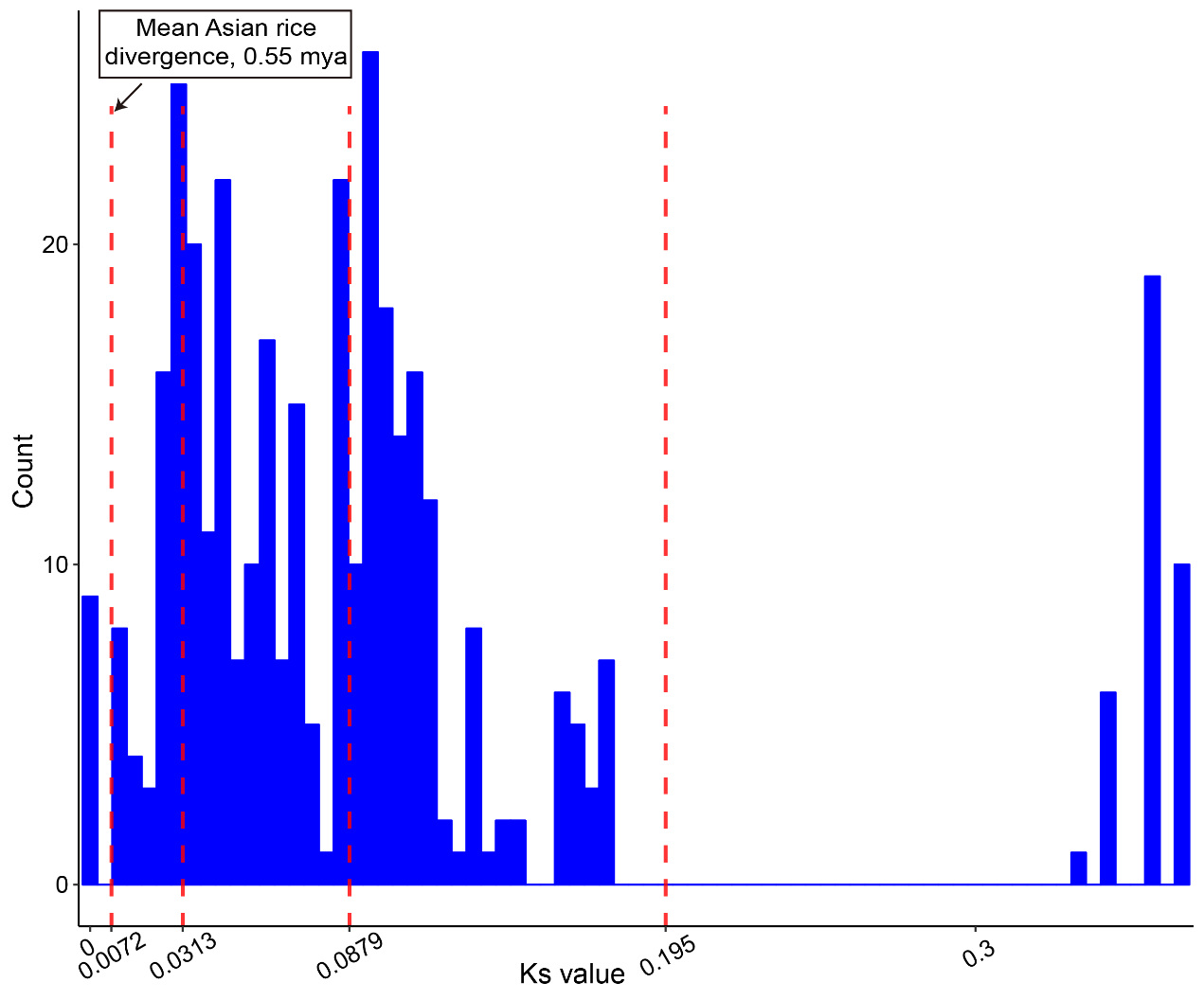
**

#### **Figure S9. The Ks distribution of major-minor duplicated pairs.**

Most of the major-minor duplicated pairs could be traced back to before the divergence of Asian rice (> 0.55 mya, Ks > 0.0072). The four Ks valuses (red dotted line marked) represent four key evolutionary events in the evolution of the *Oryza* genera, respectively, referring in the Stein et al (2018).
